# Optimizing Sampling and Extraction Methods for Plant-Parasitic and Entomopathogenic Nematodes

**DOI:** 10.3390/plants10040629

**Published:** 2021-03-26

**Authors:** Mahfouz M. M. Abd-Elgawad

**Affiliations:** National Research Center, Plant Pathology Department, Agricultural and Biological Research Division, El-behooth St., Dokki 12622, Egypt; mahfouzian2000@yahoo.com

**Keywords:** index of dispersion, IPM, modelling, molecular approaches, sampling and extraction

## Abstract

Plant-parasitic and entomopathogenic nematodes (PPNs and EPNs) are key groups in crop production systems. This study aims at optimizing nematode sampling and extraction methods to benefit integrated pest management (IPM) through (a) management of PPNs and (b) use of EPNs. The impacts of these methods on PPNs and EPNs to achieve cost-effective and efficient IPM programs are presented. The common misuses of sampling and extraction methods are discussed. Professionals engaged in IPM should consider sampling the reliability level in the light of the intended goal, location, crop value, susceptibility, nematode species, and available funds. Logical sampling methodology should be expanded to integrate various factors that can recover extra EPN isolates with differential pathogenicity. It should seek for the best EPN-host matching. Merits of repeated baiting for EPN extraction from soil and sieving for PPN recovery from suspensions are presented. Their extraction values may be modelled to quantify the efficiency of nematode separation. The use of proper indices of dispersion to enhance the biocontrol potential of EPNs or save costs in nematicidal applications is ideally compatible with IPM programs. Selecting an extraction method may sometimes require further tests to find the best extraction method of the existing fauna and/or flora. Cons and pros of modern sampling and extraction techniques are highlighted.

## 1. Introduction

Plant-parasitic and entomopathogenic nematodes (PPNs and EPNs) represent two key groups as damaging [1] and beneficial [2] organisms, respectively in crop production systems. Their sampling and extraction methods should be optimized to benefit integrated pest management (IPM) through (a) management of PPNs and (b) use of EPNs. Addressing both groups might be a little tricky but the soil is their original habitat. They have a few sampling and extraction issues related to assessing their populations, distribution patterns, and interactions with many other factors within the context of IPM.

## 2. Sampling Goal and Conceived Scenario

As sampling pertains importantly to every aspect of nematode study and management, its significance and drawbacks will cover all related scopes. Sampling of PPNs is basically intended to detect, identify, and estimate their population densities in soil or plant tissues. Its timing, pattern, intensity, tool, and the associated material sampled, all depend on the desired goal, carefully conceived scenario to avoid problems and allocated funds. For example, heavily nematode-infected plants may consequently possess too small a root system to support many PPNs, whereas samples from nearby less infected plants may harbor more nematodes for relatively large root system. Soil samples preferably obtained from the rhizosphere are often used to count PPN number per unit (either volume in cm^3^ or weight in g, but it is quite better in this case to express nematode number per g of feeder roots in the same volume of soil. This is especially important to avoid discrepancy of PPN population densities relative to plant damage. Clearly, this issue will result in a false correlation between nematode population levels and plant growth parameters/yields based on using either volume or weight unit, not both.

Sampling may also be utilized in a survey, advisory service, research, or relating population level to specific biological/ecological factor(s) or production practices. Plant root, instead of soil samples, may sometimes be good alternatives. Even one individual of any root-knot nematode (RKN) *Meloidogyne* species in a root sample of a highly susceptible crop, may call for PPN control measure, be it (regulatory, cultural, and sanitary methods, nematicide, rotation, resistant variety); e.g., according to the number of RKN-galls, more than one of these control methods may be properly used in IPM [1,3]. This applies for most species/varieties of solanaceous (tomato, eggplant, potato, pepper) and cucurbitaceous (melon, watermelon, squash, pumpkin, zucchini, cucumber) crops. For advisory service, soil and roots should be sampled for PPNs at planting or pre-planting of annual crops.

Sampling to relate the nematode population level to specific biological/ecological factor(s) or production practices can effectively contribute in IPM. It can monitor population level and impact of any biological control agent (BCA) for its further development. It can also detect harmful organisms to prevent their suppression of any beneficial invertebrate [4] in IPM. In such cases, sampling may be done just at the planting and harvest times. However, more informative sampling times may be better. It would preferably fit different growth stages of the plant. This enables pest control operators to know whether prevalence of natural or introduced BCA gradually or rapidly decline with each stage. This approach clearly addresses IPM for both PPNs and EPNs. It may monitor BCAs (e.g., EPNs against insect pests or fungi/bacteria against PPNs) for different IPM programs.

## 3. Ecological Considerations and Concepts

It is well known that there is inherited sampling error, but the most accurate samples should be obtained from locations and at times when population size is greatest in general [5,6]. Inaccuracy of assessing nematode population level is known as sampling error. Precision/reliability is the probability of getting a specified degree of sampling accuracy. Both should be considered for sound IPM. Sampling reliability is used either in terms of the standard error to mean ratio (*E*) or the ratio of the half-width of the confidence interval to the mean (*D*) of the samples [7,8]. The reliability level acceptable as a basis for PPN control in IPM decisions may vary due to the location, crop, nematode species, and available fund or personnel.

Sampling should be done to get accurate data on the pest’s ecology for effective IPM. Its design and timing should also enable us to grasp BCA ecology and biology as well as host-BCA interactions. Edaphic and crop factors (e.g., soil properties, cultivar susceptibility, nematode-economic threshold, planting, harvest times, and previous crops), and climatic factors (rainfall, temperature, humidity, solar efficacy) may add better perception for the used IPM strategy. These variables can reveal the positive or negative role of a specific production practice in IPM. Generally, pesticide usage, tillage, crop rotation, and fallow periods can adversely disrupt BCA populations [9]. Biological control of PPNs using an introduced BCA may not be as effective in various settings as that of indigenous BCA due to ecological validity. Soil moisture and texture [10], salinity [11], mulching [12], and pH [13] were also found to modulate EPN populations directly or indirectly by influencing their hosts or enemies [4].

Though often used, random soil samples suffer from the possibility that samples may chance to target an unimportant range of biotic and edaphic factors. So, most soil nematodes and related organisms remain unsampled. In contrast, stratified random sampling can upgrade efficiency to assess population densities and related factors if variations in a stratum are obviously less than that among strata. So, dividing the strata should be based on factors known to the farmer; e.g., difference in soil characteristics, productivity of previous crops, or susceptibility of these crop varieties to PPN-infection. Stratified random sampling may not only offer better estimate of PPN population levels but can also lower pest-control cost via IPM of individual or uniform strata. Regular zigzag patterns with dimensions smaller than the nematode foci can adequately sample PPNs and offer proper weight to the larger, non-infested area as nematodes mostly have clumped distribution [7]. Such patterns can more accurately assess the population density than random sampling especially when more sampling points are taken across plant rows than within rows [5].

Nematode extraction should consider the related settings and nematode genera. For example, extraction of nematode cysts (genera Heterodera and Globodera) may differ from that used for *Meloidogyne* spp. [6,14]. Proper extraction techniques should best fit the existing organisms (e.g., protozoa, fungi, bacteria, invertebrate predators, omnivores, and microarthropods) as the extraction efficiency varies among species. Sucrose centrifugation is the most efficient method for microarthropod extraction. It is) used [15] as a model to study nematodes and their natural enemies such as collembolan and acari mites. Such techniques as species-specific primers and probes in quantitative polymerase chain reaction (qPCR) assessment, colony culture to count colony forming units per unit of root weight, sucrose centrifugation, Baermann funnel, sieving, or baiting with EPN-susceptible host may vary in the extraction efficiency [6,14,16].

Various factors can share in cost-effective and efficient IPM programs. Cost can be reduced via more efficient sampling procedures. A common mistake is to assume that there is always a linear relation between sampling cost and sample size. The variation due to laboratory procedures in the sampling and extraction methodology are mostly unknown and may even exceed field variation that requires more samples. One would rather improve methods instead of reducing samples. A big gap in the accuracy and precision of nematode counts resulting from inter-laboratory proficiency tests was reported [17]. The reasons may be the different custom-made equipment, laboratory-specific adaptations, and/or relative operator’s experience. Manufacturers of sampling and extraction tools should continuously contact the related stakeholder for tools’ fine-tuning and upgrading. The tests should further be expanded to evaluate and fix the quality of the laboratories’ own methods especially in developing countries. This will help to gain insights into possible trends and potential refinements. Mechanized sampling could improve the accuracy and precision, but it requires well-qualified operator on the mechanical sampling equipment (e.g., operate the tractor in a sound and safe manner, sound review for the map of sampled area and handling of the samples, bags, and bag holder).

## 4. Sampling Tools

**Figure 1:**
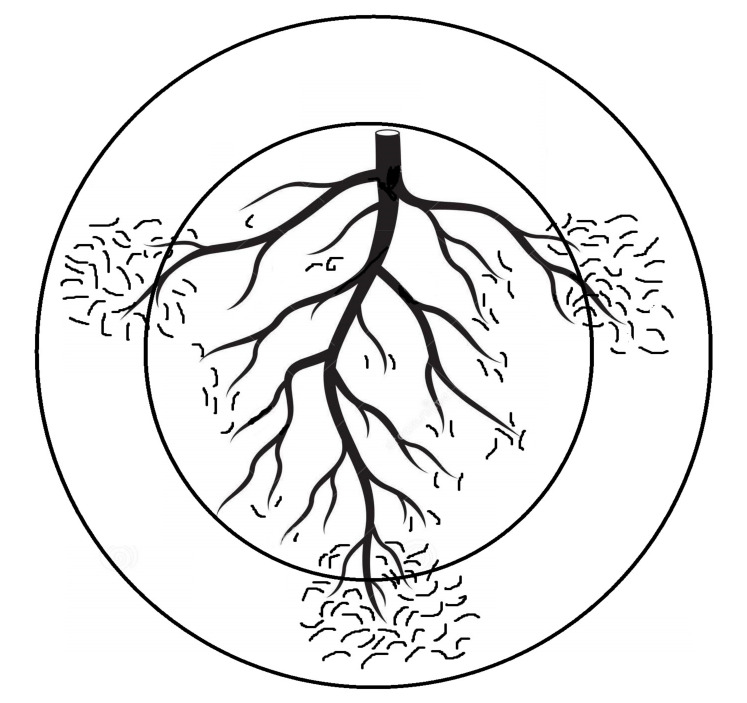
Two quadrat sizes are represented by concentric circles. The inner circle represents random nematode distribution around plant main root and the outer circle represents clumped nematode distribution around lateral fibrous roots as well.

Conventional soil samplers [14] such as augers to obtain cores are often used in developed countries while an ordinary spade, bladed shovel, or hand trowel is frequently used in developing countries. The use of these variable samplers for similar IPM programs may lead to erratic results. The difference in volume/area of the sampling units may influence the obtained distribution patterns of the pest or BCA [18]. Though acceptable, they lack in the standardization of the used sampler which may falsely contribute to the value of the same index of nematode dispersion used (Table 1). Even sampling for similar objectives is taken with cores that may differ in diameters (e.g., 17, 18, 20, and 25 mm) from one trial to another. This may lead to inconsistent results and misinterpretation of data. For instance, sampling the same site with two concentric circles (as core or unit area) might unexpectedly reveal different spatial patterns of the same population. These patterns (Figure 1) are so different that the nematode counts would require log (for aggregated distribution) or square root (for random distribution) data transformations to equalize experimental treatment variances; a pre-requisite to use parametric statistical methods such as analysis of variance, regression, and correlation [19]. Moreover, adopting a standardized sampler can grant sound comparison between different trials and expand analysis of individual trials for perfection of the conclusions. For vertical distribution, deep-rooted crops require deeper sampling (e.g., grape; 60 cm depth) than shallow-rooted ones (e.g., squash; 20 cm depth) but generally a depth of 30 cm can target the nematodes in the zone of their highest density. A standard core of 2 cm diameter with adjustable depths may be suggested unless it stifles an innovation or experimental goal. Notably, this suggestion avoids other drawbacks because characteristics of a distribution pattern are often dependent on the “standard” scale over which it is processed. Manufacturers and suppliers of such tools would preferably consult pest control operators to standardize their products for better IPM.

## 5. Addressing Nematode Distribution Patterns

These patterns, revealed by sound sampling, can enable pest control operators to: (1) choose plant material that best fit to specific locations, (2) leverage variable rate methods for the used nematicides, and (3) characterize relationships between organisms in space and time for careful IPM. Stuart and Gaugler [25] stressed that nematode clumped distribution can have great ramifications at the community levels by changing the dynamics of parasitism, predation, and competition. Such spatial (horizontal or vertical) and temporal distributions may be compared with one or more of the relevant biotic and physical forces for better development of IPM. Moreover, definite models [26,27,28] (4) can serve in the nematode-count transformation to fulfill accurate treatment comparisons.

However, samples often become costly to offer these merits of distribution patterns. So, a trade-off between objectivity and cost is necessary. For convenience, recent trends offer different accuracy levels for the same sample size and tactic to meet affordability. Iteration was also used to further improve optimum sample size [29]. Increasing the cost by increasing the number of cores or samples, or both, should be weighed against the benefit it provides via accuracy and reliability [30]. Collecting more small cores, though costly, offers a more accurate mean estimate than an equivalent amount of soil collected as fewer large cores [5]. Moreover, collecting numbers of cores/sub-samples from a targeted area before mixing into a composite sample to homogenize variance of nematode counts may reduce costs though it introduces another potential source of statistical variability. A modification is to take subsample(s) from the composite sample to estimate the population density and/or the numbers of BCA/endospores associated with the nematodes per sample to reduce costs.

## 6. Indices of Nematode Dispersion

We should cautiously suggest type and number of these indices to fit the goal of the work. For instance, contrary to Taylor’s Power law (TPL) [31], Spatial Analysis by Distance Indices (SADIE) has geographic coordinates. [32] reviewed geostatistical models as another group that can apply sample values and locations simultaneously to depict spatial patterns and estimate values at unsampled locations. Yet, this group does not offer tests to assess the statistical significance of the patterns but SADIE software can determine the statistical probability level of spatial association between organisms or the same organism at different times [33]. So, these indices can complement each other to show more aspects of the distribution patterns. Gorny et al. [34] manipulated two indices to set sound sampling protocols and determined specific sites for nematicide usage. Moreover, Wu et al. [23] used SADIE to prove regulation of EPNs by a natural enemy. Therefore, the use of such indices to enhance biocontrol potential or to save costs in nematicidal applications is ideally compatible with IPM.

Conceivably, nematode spatial patterns are more representative in samples taken far apart which will be more impacted by various microhabitats than samples taken close to each other. This concept could be backed by using both semivariogram and SADIE analyses together to better grasp PPN and/or EPN spatial patterns and spatiotemporal dynamics [34,35]. To facilitate its use, SADIE program in terms of its major indices and graphical displays were recently reviewed [36]. It was integrated with other methods to study soil food webs in citrus orchards in order to develop new biocontrol approach that can serve in IPM [32,37].

Complementary methods [38,39] can optimally detect spatial heterogeneity when clusters are situated on elongated or square domains and near to the edges of the surveyed sites. They can reveal clusters with small radius and in sample size smaller than that of SADIE as well as adjust for the absolute location or the magnitude of the counts.

## 7. Other Examples to Optimize EPN Sampling and Extraction

A main challenge facing the use of EPN is to broaden the EPN species/strain library in order to provide suitable matches of nematodes to target pests. This will certainly optimize their benefits as biocontrol agents. The wide variation of EPN sampling makes results from a definite case-study difficult to generalize. Nevertheless, it is quite evident that the percentage of samples positive for EPN in many typical surveys worldwide are relatively low; <35% [12,40]. There is a dire need to increase it to likely offer new strains and upgrade EPN-host matching. So, novel sampling concepts to get EPN with high recovery frequency value and differential pathogenicity should be further sought. One such recent concept relies on combining four factors. The factors are favorable sampling method, time, site targeting, and use of multiple extraction technique. This combination could recover EPN from the seven surveyed groves and from 61.7% soil samples [24]. On the contrary, only one EPN-positive out of 593 soil samples was detected also in Egypt [41]. However, they used random sampling and single baiting cycle. Moreover, the EPN isolates recovered via rational sampling showed so variable pathogenicity to the strawberry white grub, *Temnorhynchus baal* [42]. Using such criteria or other new concepts to optimize EPN sampling and recovery frequency value should be further tested and expanded.

Another example is the invasive mole crickets as major pests of pastures, turf, and vegetables in the Caribbean Basin where *Steinernema scapterisci* is the only EPN species utilized efficiently as classical biocontrol strategy. It is used against the mole crickets [43]. Classical biocontrol should be expanded via directing sampling and extraction techniques to isolate BCA from environments where the organisms will presumably have had to develop the desired trait [44].

Specifically, the extraction technique using multiple *Galleria*-baiting cycles proved more effective than a single cycle in several studies [25,28,45]. Moreover, stressing by crowding, abiotic/biotic factors in soil, or presence under other suboptimal conditions may prevent or delay the nematode activity for infection [46,47]. Optimizing conditions for infection may gradually revert the EPN activity to infect the baiting insect in a consecutive cycle. Repeated extraction via baiting cycles can usually provide optimal conditions and longer time, for such a revision. Hence, it allows for differential pathogenicity of EPN too. A common technique is to keep the soil samples at about 23 °C in suitable cups with 4 last instar *Galleria mellonella* larvae as baits per cup in each cycle. Soil is sometimes watered to remain almost at field capacity during the extraction cycles. Each cup is inspected twice weekly in the first 3–4 weeks but once thereafter. Each cycle ends by inspecting the cups to: *i*) isolate insect cadavers with symptoms of EPN infection. These cadavers are transferred to White traps [48] to fulfill Koch’s postulates, and/or *ii*) discard the other dead insects. A new following cycle begins with replacing the infected cadavers/dead insects by new living *G*. *mellonella* larvae. Suspect cadavers that failed to produce EPN-infective juveniles (IJs) are considered negative. The first cycle may be repeated 5–10 times [25,28] depending on the magnitude of EPN-positive samples. Other modifications to improve the baiting method are possible. They may include screening for EPN by using the target insect pest species; e.g., citrus root weevil, *Diaprepes abbreviatus* [49] or pecan weevil, *Curculio caryae* [50], as baits to achieve adequate EPN-host matching. Moreover, two model insect species/baiting at different temperatures to increase and diversify the recovery of EPN were tried [50,51]. These trends to find ecologically adaptable and effective BCAs should not be limited to a specific region or pest. Biocontrol components can strengthen IPM programs by using indigenous, or to a less degree introduced, EPN against the target ‘baiting’ pest or via setting the best EPN-host matching.

## 8. Other Sampling and Extraction Methods

EPNs in soil may be detected directly under binocular microscope via dissecting or enzymatic hydrolysis of the EPN-infected-cadavers or indirectly by scoring the cadavers per sample. Other methods of extracting EPNs from soil or their host insects [16,52] and PPNs from soil or plant tissues [5,6,7,14] were reviewed. Pest control operators must consider their relative merits and demerits for perfection of IPM. For instance, sieving and centrifugation using a sucrose gradient may directly extract and quantify dead and live EPN-IJs and PPNs from soil samples. The method may recover a larger proportion of EPNs in soil than insect baiting. It is less biased due to differential pathogenicity among EPNs extracted via the baiting method. However, it is rarely used to recover EPNs as it is more labor-intensive and require taxonomic expertise for the recovered nematodes [16]. Baermann funnel method and its modifications can extract only live nematodes. Selecting a method may sometimes require further tests to find the most efficient extraction method of the existing fauna and flora related to IPM [15].

## 9. Quantifying Extraction Efficiency of EPNs with a Model Used for PPNs

**Figure 2:**
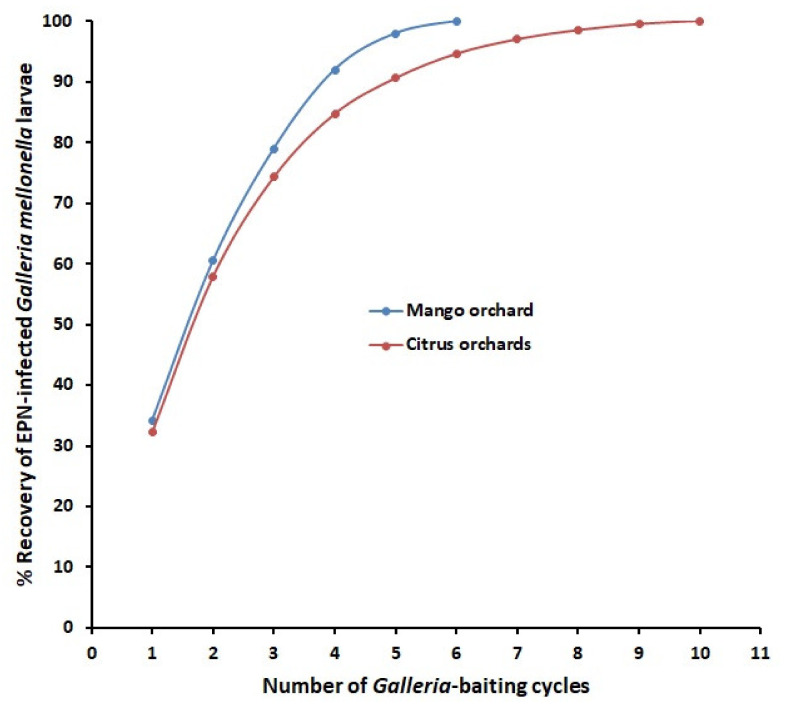
Calculated relationships between number of *Galleria*-baiting cycles and percentage recovery of entomopathogenic nematodes-infected insects for surveys of mango and citrus orchards.

Nematode extraction via sieving, mostly favored for PPNs. or insect baiting, often used for EPNs, is based on physical (aperture sizes of the sieves) or biological (susceptibility of the baiting insects) background, respectively. So, it is exciting to find out their extraction efficiency herein via modeling. To test efficiency of sieving processes, the PPN suspension is poured through a stack of like sieves, and the recovery on each sieve is assessed. So, the cumulative recovery is related to number of times sieved. [53] related the number of sieving to percentage recovery of PPNs in the formula: Percentage recovery = 100 (1 − *a^x^*) where *a* is proportion of total number of PPNs present of a given length which pass through the sieve, and *x* is number of times sieved. The equation is used herein for EPN extraction too where *a* is the proportional loss at each *Galleria*-baiting cycle, and *x* is the number of repetitions (baiting cycles). The raw data of two EPN surveys were applied to the formula where 6 [28] or 10 [24] *Galleria*-baiting cycles produced positive samples (Figure 2). Herein, the practical % recovery of EPNs vs. theoretical corresponding values were 79% vs. 99% and 74.3% vs. 98.3% for % recovery of EPNs from mango [28] and citrus [24] orchards, respectively (Figure 2). This formula may offer approximate quantification of separation efficiency during the extraction processes. It allows consideration of the benefit to be gained by devoting more time and resources into the used EPN separation techniques [14,54] for IPM.

## 10. Molecular vs. Traditional Sampling and Extraction Technology

Limitations of traditional sampling and extraction methods are apparent. Notwithstanding the utility of a series of extractions using the above-mentioned methods to significantly enhance the PPN- and EPN-separation efficiency, they do not provide a full recovery rate [6,45]. Moreover, not all EPN species can be isolated using just one insect bait species [55]. The most common *Galleria*-baiting method can hamper the laboratory maintenance of certain EPN species (e.g., *Steinernema kraussei*).

Sampling and extraction of biochemicals are relatively newer approaches. Relevant assays [6,16] may extract proteins or isozymes from the nematodes (e.g., for identification) or from their hosts (e.g., for measuring enzyme activity of a host species/cultivar related to its compatible or incompatible reaction to nematode infection). These accurate assays may designate PPN susceptible or resistant plant cultivars and assess the contribution of BCAs in priming the plant against PPNs [56]. Extraction of isozymes has enabled the study of species diversity, frequency, and abundance to study the nature conservancy and biodiversity. Moreover, new isozyme phenotypes may be detected particularly in conserved areas that may thrive our grasping of biogeography and ecology of key species such as RKNs [57]. Moreover, sampling methods to detect and measure volatiles in the soil atmosphere in situ can enable the study of chemical cues that are critical to communicate across various trophic levels of different organisms. Hence, they can assist in grasping the IPM scenario in the soil [32]. However, reliable results can often be obtained with nematodes at a specific developmental stage.

In contrast, DNA-based diagnostics do not rely on the express products of the genome and are independent of environmental influence or developmental stage [6]. Significant gains in sampling and extraction of nematodes and their related organisms are in progress due to introducing the polymerase chain reaction (PCR) and relevant techniques [6,19,58]. The relationship between the EPN numbers in soil samples extracted by conventional techniques and the numbers recovered via qPCR approaches could be established by [59] as a base to count EPN via the molecular technique. The novel set of primers and probes integrated with the qPCR systems could then optimize a protocol for extracting nematodes and DNA from soil samples. The protocol can detect even one EPN added to a nematode community [60]. This method could detect and quantify soil-inhabiting organisms (EPNs and their related nematophagous fungi, ectoparasitic bacteria, and competitor free-living nematodes) in Florida citrus groves and examine the EPN soil food web in various ecological settings [16]. Campos-Herrera et al. [61] used qPCR to reveal sympatric distributions of EPN species and detected their low numbers in samples where the insect baiting method failed.

These molecular tools were integrated with appropriate models, e.g., indices of dispersion, in order to: (1) clarify soil food webs that modulate the rates of a herbivore-disease complex [37], (2) prove regulation of EPNs by a natural enemy where manipulating a soil property (pH) can enhance biocontrol of an insect pest [4], and (3) examine geospatial relationships between native EPN and the fungus *Fusarium solani* in citrus habitats [23]. Such gains can enable us to better conceptualize biological control potential of pests and pathogens within sound IPM context.

New molecular methods are still in the pipeline or are of limited geographic scale. Using species-specific primer-probe combinations and the high throughput sequencing [62] to characterize nematode communities and their natural enemies in soil are often used in developed countries. These methods are generally costly and require a variety of reagents and equipment of medium-high technology levels that are rarely produced in developing countries. Their cost issue will exacerbate if the local currency has gone a drastic exchange rate. A current limitation is that qPCR will identify and quantify only those organisms for which the molecular toolkits are employed. It does not reveal the presence of those species not screened for, or species for which the qPCR was not developed. Therefore, in areas where EPN diversity is not well known, the insect-bait method is done to isolate new and/or unexpected species [16]. The insect-baiting can detect new species and provide their activity (ability to kill) data.

The primer/probe combination is designed to be specific for a single species, but discovery of closely related species in the sampled area might increase the likelihood of cross-amplification. So, optimizing the approach in a new system is recommended. It requires great skill in molecular biology. If not, contradictory results may be due to imperfectly carried out tests. Moreover, contamination of the used reagents may indicate false positives for some EPN species. In this case, re-sampling and repeating the tests will be required and increase the costs. Finally, qPCR and insect-baiting may or may not agree. The qPCR method indicated high numbers of IJs, but no insects were infected when the same soil was baited [63]. So, more studies are needed to trust the merits and demerits of each technique (qPCR and insect-bait).

In parallel to EPN, adequate methods for DNA extraction from the PPNs and related BCA are going ahead. For instance, techniques using beacon probe qPCR to detect, quantify, and surveil PPN antagonists in samples are applied. Regaieg et al. [64] used this technique to evaluate capability of the fungus *Verticillium leptobactrum* to colonize RKN-egg masses. Its accurate quantification of the *V. leptobactrum* DNA over the egg masses can help in unraveling the complexity of the soil ecology that has many biological and physical factors. These methods can identify pathogens such as PPNs and discriminate resident microbial populations and cells or propagules which form the released BCA [65]. Isolation of BCAs and genomic DNA extraction from the organisms are described elsewhere [66]. The methods are ideally used collectively; combining morphology, biochemical, and molecular attributes of the organism. This strategy is necessary to strengthen diagnose, define species boundaries, and offer a comprehensive database for BCA and PPN species that can serve IPM programs [57]. Multiplex PCR can detect one or several species in a nematode mixture by a single PCR test, thus decreasing diagnostic time and costs. Cautious must be exercised in this technique for identifying several nematode targets in one assay. It is limited by the available primer pairs that can be used in a reaction and the number of bands that can be identified without giving false-positive results [6]. It requires precise optimization of the reaction conditions for the primer sets used simultaneously in the test.

In conclusion, advances in IPM programs related to nematology can be achieved via optimizing sampling and extraction methods. Solving their related issues via perseverance will lead to gain more experience and refine current methods. The price of related devices on which new technologies are based usually drops rapidly after a short marketing time. So, it is expected that decreasing costs for sequence analyses will allow its wider application for diagnostics and quantification of nematodes and related organisms. This optimism will serve IPM programs concerning nematodes in many ways such as unravelling the complexity of nematode interactions in soil and characterizing their food webs, taxonomy, and best EPN-host matching.

## Figures and Tables

**Table 1:** Comparison of index of aggregation (I_a_)* values of five studies on entomopathogenic nematode [EPN] distributions using different sampling approaches in various regions.

EPN Studied Population	Form of the Measured EPN	I_a_ Value	Comments (Location)	Reference
*Heterorhabditis bacteriophora*-infective juveniles (IJ) applied uniformly, in one, or nine patches on Kentucky bluegrass	EPN-infected *Galleria mellonella* larvae over time	All mean values were less than one but differed (*p* ≤ 0.05) until 20 weeks, no more, after EPN application	The values suggest a more even distribution than a random one (NJ/USA)	[20]
Natural populations of *Steinernema feltiae* and *S. affine* in grassland plots	IJ assigned to one of 4 groups of increasing physiological age	The values ranged 1.27–1.45	All values indicate aggregated distribution (Merelbeke/Belgium)	[21]
*H*. *bacteriophora* or *S*. *carpocapsae*-infected *G*. *mellonella* larvae applied within 24 h of initial IJ emergence to cultivated fields and adjoining grassy border plots	*H*. *bacteriophora* and *S*. *carpocapsae*-IJ recovered from *G*. *mellonella* larvae baits applied several times after the cadavers	Range <1 to >2. Mean values differed between EPN species in bait traps and between soil management regimes at 48 h and 16 days after placing the cadavers, respectively	Spatial distributions dispersed from a grassy border to the adjacent cultivated field plots were more aggregated for *H*. *bacteriophora* than for *S*. *carpocapsae* (OH/USA)	[22]
*Steinernema diaprepesi*, *Heterorhabditis indica*, and *Heterorhabditis zealandica*	EPN were measured using quantitative qPCR during a 6-month citrus orchard survey	The values ranged 0.8–1.3 over 6 months and could be compared with those of the fungus and Diaprepes root weevil	Highly significant spatial associations between *Fusarium solani* and EPN communities of up to three EPN species (FL, USA)	[23]
Natural populations of *H*. *indica* in citrus groves	EPN-infected *G. mellonella* larvae	0.913	I_a_ refers to uniform distribution pattern (Giza, Egypt)	[24]

I_a_ = the observed value of distance to regularity/the mean randomized value [25]; qPCR: quantitative polymerase chain reaction.
